# Relationship between Bruxism and Malocclusion among Preschool Children in Isfahan

**DOI:** 10.5681/joddd.2012.028

**Published:** 2012-12-11

**Authors:** Maryam Ghafournia, Maryam Hajenourozali Tehrani

**Affiliations:** Assistant Professor, Department of Pediatric Dentistry, School of Dentistry, Isfahan University of Medical Sciences, Iran

**Keywords:** Bruxism, occlusal factors, preschool children

## Abstract

**Background and aims:**

Bruxism is defined as a habitual nonfunctional forceful contact between occlusal tooth surfaces. In younger children bruxism may be a consequence of the masticatory neuromuscular system immaturity. The aim of this study was to assess the prevalence of bruxism and investigate the relationship between occlusal factors and bruxism among preschool children.

**Materials and methods:**

In this cross-sectional survey, 400 3-6-year-old children were selected randomly from different preschools in Isfahan, Iran. The subjects were divided into two groups of bruxers and non-bruxers as determined by the clinical examination and their parents’ reports. The examiner recorded the primary canines (Class I, Class II, and Class III) and molars (mesial step, distal step, flash terminal plane) relationship, existence of anterior and posterior crossbite, open and deep bite. Also, rotated teeth, food impaction, sharp tooth edges, high restorations, extensive tooth caries, and painful teeth (categorized as irritating tooth conditions) were evaluated. The relationship between bruxism and occlusal factors and irritating tooth conditions was evaluated with chi-square test.

**Results:**

Bruxism was seen in 12.75% of the subjects. Statistically significant relationships existed between bruxism and some occlusal factors, such as flash terminal plane (P = 0.023) and mesial step (P = 0.001) and also, between food impaction, extensive tooth caries, tooth pain, sharp tooth edge and bruxism.

**Conclusion:**

The results showed significant relationship of bruxism with primary molar relationships and irritating tooth conditions among preschool children.

## Introduction

Bruxism, defined as a habitual nonfunctional forceful contact between occlusal tooth surfaces, is involuntary, excessive grinding, clenching or rubbing of teeth during nonfunctional movements of the masticatory system. Bruxism may occur during the day or during sleep.^1^ Tooth grinding that usually occurs during sleep has been named nocturnal bruxism and is linked to cranio-mandibular disorders including headaches, temporomandibular joint discomfort, muscular pain, premature loss of teeth due to excessive attrition and mobility, and sleep disruption of the individual as well as that of the bed partner.^2-4^

The etiology of bruxism has been attributed to systemic factors, such as intestinal parasites,^5^ subclinical nutritional deficiencies, allergies, and endocrine disorders^6^ and also to local factors, especially malocclusion^7,8^ or supernumerary tooth^9^ and to psychological factors.^10,11^ Genetics also plays a role.^12,13^

It is generally believed that sleep bruxism is more common during childhood, although it is not uncommon in adults and occurs less commonly in the elderly.^14^

In recent years, bruxism has become an increasing concern in children due to its negative effects on life quality and also for being considered an important risk factor for temporomandibular dysfunctions. It may cause tooth wear and, in more severe cases, dental traumas.^12,15^ Recent studies have indicated a relation between bruxism and respiratory alterations.^16-18^ Other implications for orofacial maturity and speech are still not well established.

Moreover, in younger children, bruxism may be a consequence of the masticatory neuromuscular system immaturity.^15^

The prevalence of signs and symptoms of temporomandibular disorders are described in the literature for various worldwide ethnic groups. In children, bruxism is becoming an increasingly common condition.^11^

While Serra-Negraet al^19^ used only a questionnaire to determine its prevalence of 35.3% in children, Fonseca^20^ assessed the prevalence of bruxism to be 15.29% with a short questionnaire and a clinical examination for wear facet.

Taken together, few studies have attempted to survey the occlusal factors involved in bruxism. It would be useful to know this relationship in order to prevent bruxism developed by occlusal disorders.

Several studies have assessed the relationship between bruxism and malocclusion among children, with reports of the significant relationship between bruxism and some occlusal factors.^21-23^ However, Demir et al^24^ and Cheng et al^25^ showed that some of the occlusal factors were not seemingly involved in the development of bruxism.

Oral parafunctions in childhood might be a persistent trait in many cases. Abnormal occlusion and tooth wear in childhood predicted increased anterior tooth wear 20 years later.^26^ Therefore, it is imperative that pediatric dentists be informed about the current lines of evidence regarding bruxism in children. In management of this parafunctional habit, clinicians play a pivotal role to determine the possible etiological factors. In many situations it is the dentist’s task to warn parents and plan a multidisciplinary treatment.

The aim of this study was to determine the prevalence of bruxism and investigate the relationship between occlusal factors and bruxism in 3-6-year-old preschool children in Isfahan.

## Materials and Methods

In this cross-sectional survey a total of 400 children (3-6 years of age) were included randomly from several preschools of Isfahan Province in Iran.

### Exclusion Criteria

Children whose parents were not aware of their child’s bruxism, individuals with systemic disorders, cerebral palsy, mental retardation, and children whose permanent molar teeth had been erupted were excluded.

Subjects were divided into two groups as bruxers or nonbruxers based on the child’s parent report and clinical examination based on the method described by Demir et al.^24^

The parents were questioned about their child’s bruxism, which was recorded as present or absent. The bruxing patients were required to meet all the following predetermined criteria in order to be included in this study: (1) heard to brux by parents; and (2) confirmation of bruxism by the principal investigator with an intraoral examination (presence of wear facets on teeth: grade 0, no wear facets; grade 1, enamel only; grade 2, enamel and dentin; grade 3: wear of the cusp). The nonbruxing samples were recruited based on the parents’ reports, indicating no history of bruxism , as well as an intraoral examination that ruled out obvious signs of bruxism (grade 2 or 3 of the presence of wear facets on at least four teeth).^24^

Another examiner recorded the primary canines (Class I, Class II, Class III) and molars (mesial step, distal step, flash terminal plane) relationship bilaterally, existence of anterior and posterior crossbite, and also open and deep bite.

Rotated teeth, food impaction, sharp tooth edges, high restorations, extensive tooth caries and painful teeth, which were named as irritating tooth conditions, were also recorded.

Crossbites were designated as either present or absent. Anterior and posterior crossbite was also recorded.

History of bruxism in parents was evaluated based on their self-reports and clinical examination by methods mentioned above.

All the statistical analyses were performed using the SPSS software package (SPSS for Windows 7, Version 10.0, SPSS Inc, Chicago, USA). The relationship between bruxism and occlusal factors or irritating tooth conditions was evaluated with chi-square test.

## Results

Bruxism was seen in 12.75% (51) of the subjects, of whom 45.01% were girls and 54.9% were boys. The mean age was 5.1 years for bruxing and 4.5 years for nonbruxing subjects. No statistically significant relationship was found between sex and bruxism (P = 0.097).

The distribution of different occlusal factors in 3-6-year-old preschool children is presented in Table 1. The results showed statistically significant relationship between bruxism, flash terminal plane (P = 0.023) and mesial step (P = 0.001).

**Table 1:** Prevalence of different occlusal factors and bruxism in the studied population (n = 400)

Occlusal factors	Number (%) of cases with bruxism	Statistical significance
Anterior open bite	—	NS
Posterior open bite	—	NS
Mesial step	25 (50%)	P = 0.001*
Distal step	6 (12%)	NS
Flush terminal plane	19 (38%)	P = 0.023*
Canine Class I	46 (92%)	NS
Canine Class II	4 (8%)	NS
Canine Class III	1 (2%)	NS
Anterior crossbite	1 (2%)	NS
Posterior crossbite	1 (2%)	NS

According to the results of chi-square test. NS: Not significant. * Statistically significant.

Table 2 summarizes the prevalence of different irritating tooth conditions and bruxism, exhibiting significant relationship between food impaction, extensive tooth caries, tooth pain, sharp tooth edges and bruxism from irritating tooth conditions.

**Table 2:** Prevalence of different irritating tooth conditions and bruxism in the studied population (n = 400)

Irritating tooth conditions	Number (%) of cases with bruxism	Statistical significance
Sharp tooth edge	12 (23.5%)	P = 0.017
Painful tooth	10 (19.6%)	P = 0.012
High restoration	5 (9.8%)	NS
Rotated tooth	19 (37.25%)	P = 0.005
Food impaction	12 (23.5%)	P = 0.004
Extensive tooth caries	24 (47.05%)	P = 0.0005

According to the results of chi-square test. NS: Not significant. * Statistically significant.

In addition, there was statistically significant relationship between the history of bruxism in parents and presence of bruxism in children (P = 0.001).

## Discussion

In this study we aimed to determine the prevalence of bruxism and investigate the relationship between occlusal factors, irritating tooth conditions and bruxism among 3-6-year-old preschool children in Isfahan.

Different techniques have been suggested to record bruxism in epidemiologic studies.^27^ One technique is the evaluation of dental attrition, from direct visual observations of the mouth,^28^ from dental study casts,^29^ or from occlusal appliances. However, it is very difficult to be sure if bruxism is a result of parafunctional or functional habits.^30^ Since the occlusal surfaces are worn physiologically in the deciduous dentition, the accuracy of the use of dental attrition is controversial.^23^ Another remarkable point is the timing of attrition because there is a risk of recording no bruxism when the subjects have recently developed bruxism and may not indicate attrition.^23^ The same risk exists when bruxism process has been stopped, though attrition is observed.^31^ Also, dental wear can be caused by many factors other than bruxism.^32^ Therefore, in this study the bruxism history was obtained by a combination of methods and interviews of the children and clinical examinations by the clinician.

The prevalence of bruxism in the literature varies from 7% to 88% in children^34^ and from 5% to 15% in adults.^35^ This diversity is probably due to different methodologies of studies.

The prevalence of bruxism in a study by Chiefetzet al,^33^ in which data was collected only via a questionnaire was reported 38% in children. In addition, Zenari et al^36^ reported a high occurrence (55.3%) of bruxism that was assessed by the parent's report.

However, in other studies that assessed bruxism with both questionnaire and child’s report, the prevalence of bruxism was lower, e.g. 15.9% in a study by Fonseca et al,^20^ 13.27% by Egermark-Erikson et al,^37^ and 12.6% by Demir et al.^24^

In this study, the prevalence of bruxism was 12.75%, similar to the results of studies by Demir et al^24^ and Egermark-Erikson et al.^37^

Consistent with other studies, no statistically significant gender effect was found on the prevalence of bruxism in the present study.^24,36,37^

Several studies have shown a relationship between occlusal factors and bruxism in permanent dentition but there are a few studies evaluating the relationship between occlusal factors and bruxism in primary dentition.

Henrikson et al^38^ reported that bruxism was higher in the Class II malocclusion group when compared with the normal group, suggesting a relationship between parafunctional habits and orthodontic malocclusion. Nilner^21^ studied the relationship between occlusal factors and bruxism and reported statistically significant correlations between Class II and Class III molar relationships and bruxism.

Carlsson et al^39^ indicated that Angle Class II malocclusion and tooth wear in childhood predicted increased tooth wear in adulthood.

In contrast to the results discussed above, a number of investigators have reported that occlusal factors are not involved in the etiology of bruxism. They found no statistically significant relationship between any type of morphologic malocclusion and tooth clenching and grinding.^23-25,40,41^

It was shown in this study that two types of primary molar relationships (the mesial step and flash terminal plane) were significantly related to bruxism, which is in contrast to the results of a study by Sari et al^23^ They found no difference between any type of primary molar relationship and bruxism.

In another study, Nilner^21^ examined the relationship between occlusal factors and bruxism in 309 adolescents. A statistically significant correlation was reported between deep bite, clenching and dental wear.

Sari et al^23^ reported statistically significant relationship between an overjet of ˃6 mm, negative overjet, overbite, open bite, and bruxism in permanent dentition.

In this study no relationship was found between bruxism and anterior and posterior open bite and crossbite, in contrast to the results of studies by Miamoto C et al^42^ and José Pereira et al,^43^ indicating that posterior crossbite is directly associated with clinical manifestations of bruxism.

The relationship between irritating tooth conditions and bruxism has not been evaluated in other studies. In the present research, significant relationships were found between bruxism and food impaction, tooth caries, tooth pain, and sharp tooth edges.

Our findings indicate the importance of regular and accurate oral examination for early diagnosis of any tooth decays in children every three months, leading to the prevention of dental pain and food impaction.

By preventing such irritating causes, one can impede the process of bruxism and thus, its adverse consequences on a children’s life quality. Further longitudinal studies with larger sample sizes are recommended to assess whether there is a relationship between occlusal factors and bruxism.

## Conclusion

The prevalence of bruxism in the evaluated 3-6-year-old children was 12.75%. Regarding occlusal factors, there was statistically significant relationships between mesial step, flush terminal plane, and bruxism. Of the irritating tooth conditions assessed, food impaction, extensive tooth caries, tooth pain, and sharp tooth edges were found to have significant relationships with bruxism.
